# Seronegative rheumatoid arthritis after combination therapy with ipilimumab and nivolumab for postoperative pancreatic and liver metastases from renal cell carcinoma

**DOI:** 10.1002/iju5.12560

**Published:** 2022-11-30

**Authors:** Yuki Nishimura, Kazuaki Yamanaka, Taigo Kato, Koji Hatano, Atsunari Kawashima, Shinichiro Fukuhara, Motohide Uemura, Ryoichi Imamura, Norio Nonomura

**Affiliations:** ^1^ Department of Urology Osaka University Graduate School of Medicine Suita Osaka Japan

**Keywords:** immune‐related adverse event, ipilimumab, nivolumab, renal cell carcinoma, seronegative rheumatoid arthritis

## Abstract

**Introduction:**

Since the approval of immune checkpoint inhibitors for renal cell carcinoma treatment, therapeutic efficacy has been enhanced. However, although autoimmune‐related side effects may occur, rheumatoid immune‐related adverse events seldom develop.

**Case presentation:**

A 78‐year‐old Japanese man with renal cell carcinoma developed pancreatic and liver metastases after bilateral partial nephrectomy and was treated with ipilimumab and nivolumab. After 22 months, he developed arthralgia in limbs and knee joints, accompanied by limb swelling. The diagnosis was seronegative rheumatoid arthritis. Nivolumab was discontinued, and prednisolone was initiated, quickly improving symptoms. Although nivolumab was resumed after 2 months, arthritis did not recur.

**Conclusion:**

Immune checkpoint inhibitors may cause a wide variety of immune‐related adverse events. When arthritis is encountered during immune checkpoint inhibitor administration, seronegative rheumatoid arthritis should be differentiated from other types of arthritis, despite being less frequent.

**Keynote message**

Nivolumab and ipilimumab, employed for the treatment of metastatic renal cell carcinoma, could cause seronegative rheumatoid arthritis as an immune‐related adverse event and should be acknowledged when arthritis occurs.

**Abbreviations & Acronyms**
ACR/EULAR: American College of Rheumatology/European League Against Rheumatism
anti‐CCP: anti‐cyclic citrullinated peptide
ccRCC: clear cell renal cell carcinoma
HCQ: hydroxycholoroquine
ICI: immune checkpoint inhibitor
irAE: immune‐related adverse event
MTX: methotrexate
NR: none reported
NSAID: non‐steroidal anti‐inflammatory drug
PD‐1: programmed death‐1
PD‐L1: programmed death‐ligand 1
PSL: prednisolone
RCC: renal cell carcinoma
RF: rheumatoid factor
SASP: salazosulfapyridine

## Introduction

Since the approval of ICI therapies including ipilimumab and nivolumab, outcomes have improved in patients with unresectable cancers and metastases. However, ICI therapy is known to cause autoimmune‐related side effects that are especially prevalent in the gastrointestinal tract, endocrine system, skin, and liver.^1^ Inflammatory arthritis caused by ICIs accounts for 2.6% of irAEs. Inflammatory arthritis of the irAE type occurs frequently with ipilimumab‐nivolumab combination therapy, PD1/PD‐L1 inhibitors, and CTLA4 inhibitors, in that order.^2^ The median time of the appearance of arthritis was 70 days after initiation of ICI (range: 1–12 months).^3^ The predilection sites for arthritis are the shoulder, finger, knee, and wrist joints.^4^ Some cases of inflammatory arthritis are diagnosed as rheumatoid irAE including rheumatoid arthritis, polymyalgia rheumatica, remitting seronegative symmetrical synovitis with pitting edema syndrome, psoriatic arthritis, and spondyloarthritis. However, most cases of irAE‐associated arthritis fall into the unclassifiable category as they are often autoantibody negative and do not meet the respective diagnostic criteria. Here, we report a case where seronegative rheumatoid arthritis developed in a patient with metastatic RCC during combination therapy with ipilimumab and nivolumab.

## Case presentation

**Fig. 1:**
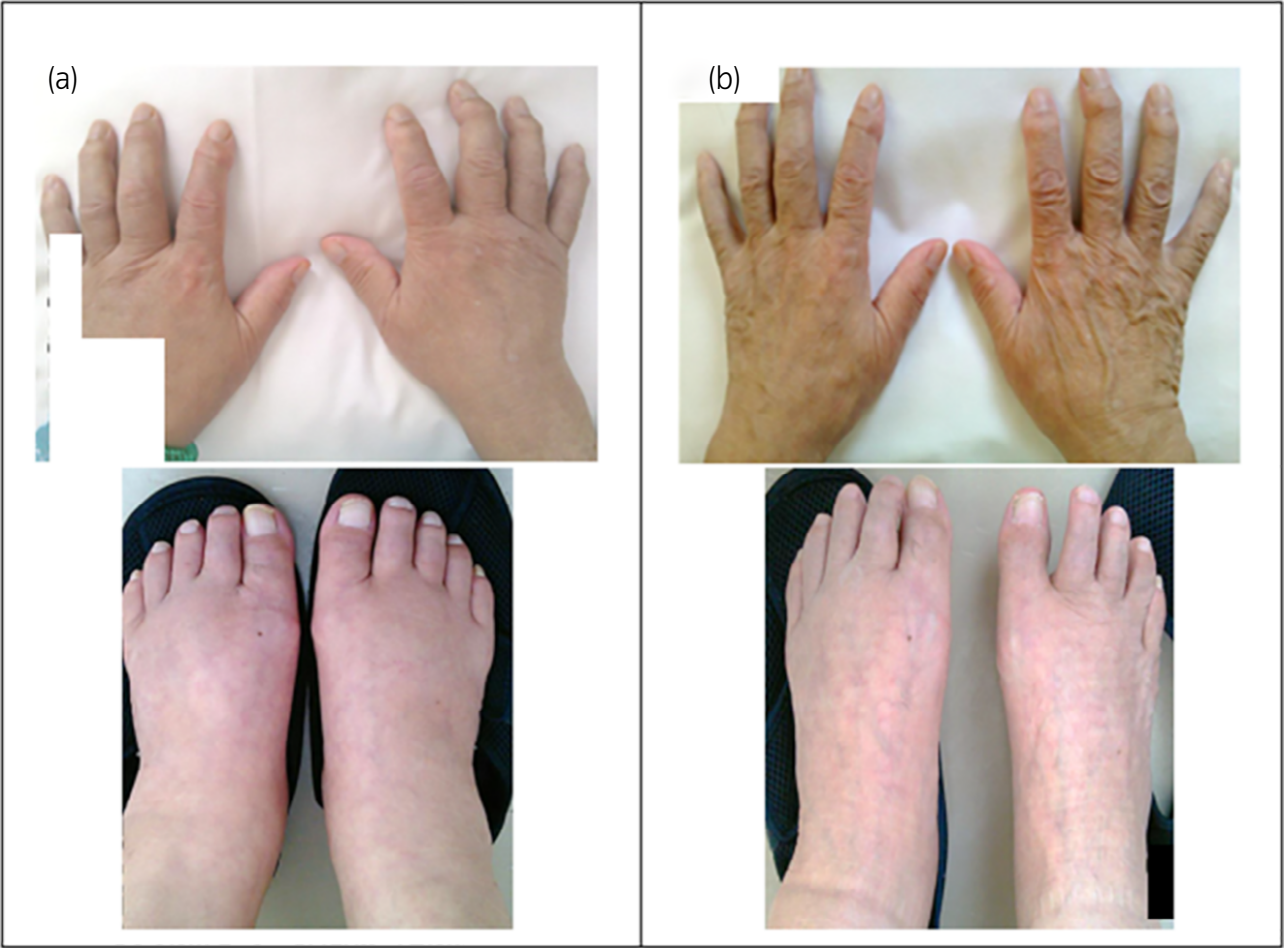
Images of the limb of the patient with arthritis before (a) and after (b) starting PSL.

**Fig. 2:**
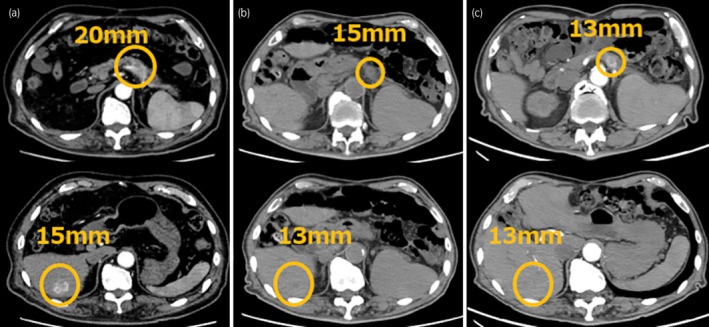
Computed tomography of pancreatic and liver metastases before administration (a), interruption (b), and resumption (c) of ICI.

A 78‐year‐old Japanese man underwent a bilateral partial nephrectomy for bilateral RCC 8 years ago. The pathological diagnosis was ccRCC. Seven years after bilateral partial nephrectomy, solitary lung metastasis appeared at the hilar region in the right lower lobe of the lung; thus, he underwent a thoracoscopic resection of the lobe, and it was pathologically diagnosed as metastatic ccRCC. Eight years after bilateral partial nephrectomy, multiple metastases to the pancreas and liver occurred. Biopsy revealed pancreatic and liver metastases from ccRCC. He started systemic therapy with ipilimumab (1 mg/kg) and nivolumab (240 mg). After 4 cycles (1 cycle/3 weeks) of combination therapy, nivolumab (240 mg) was continued every 2 weeks. Three months after initiating ICI therapy, he developed type 1 diabetes mellitus, and insulin replacement therapy was initiated. He also subsequently developed panhypopituitarism, necessitating hydrocortisone replacement therapy (15 mg daily). Fifteen months after initiation of ICI, he experienced right knee joint pain, and 22 months later, indurated swelling with edema in both limb joints also materialized (Fig. 1a). Laboratory tests indicated elevated inflammatory markers with C‐reactive protein of 9.00 mg/dL, erythrocyte sedimentation rate of 86 mm, and matrix metalloproteinase‐3 of 999.2 ng/mL. However, RF and anti‐CCP antibodies were negative, as were other autoantibodies. Ultrasonography revealed synovitis and hypervascularity in the symptomatic joints. X‐ray findings of the hands and legs presented osteophyte formations and bone erosions in 11 areas. This case was diagnosed as seronegative rheumatoid arthritis according to the ACR/EULAR rheumatoid arthritis classification criteria with a total score of 7 and negative RF and anti‐CCP antibodies. He had no past or family history of connective tissue disease or inflammatory arthritis. Nivolumab was suspended, and PSL treatment was initiated at 20 mg daily. Two days post‐treatment, limb swelling improved, and the pain vanished after 7 days (Fig. 1b). Inflammatory markers returned to normal. PSL was gradually tapered to 9 mg/day 7 weeks after initiation, and nivolumab 240 mg was restarted. The metastatic lesions in the pancreas and liver did not increase in size during cessation of nivolumab (Fig. 2). He remained stable after the resumption of ICI without recurrence of arthralgia or other irAEs.

## Discussion

ICI exerts its antitumor effect by activating an immune response against tumor cells, but it has also been implied to cause irAE due to an unwarranted autoimmune response. The NCCN Clinical Practice Guidelines specify that in cases of inflammatory arthritis with mild joint pain (Grade 1), ICI should be continued and NSAIDs or low‐dose PSL should be administered. In cases of moderate pain that limits daily activities (Grade 2), ICI should be withheld and PSL should be administered (0.5 mg/kg/day), but no conclusion has yet been drawn on the necessity of ICI discontinuation. For more severe arthritis (Grade 3) that limits daily activities or severely hinders the quality of life, ICIs may be withheld or permanently discontinued, and PSL should be administered (1 mg/kg/day). If symptoms do not improve within 2 weeks, disease‐modifying anti‐rheumatic drugs including infliximab, MTX, tocilizumab, or sulfasalazine should be included.^5^ Our patient was classified with Grade 2 and treated with steroids and ICI interruption. The decision to discontinue ICI treatment was controversial as the patient had previously developed multiple irAEs; the decision was made because of concerns regarding the risk of developing further irAEs. Sixteen cases of irAE‐associated rheumatoid arthritis have been reported in the literature, of which only three were seronegative (Table 1). Previous reports have shown that arthritis symptoms develop relatively early in seropositive rheumatoid arthritis within 6 months of ICI administration. In contrast, the time to symptom onset in the three cases of seronegative rheumatoid arthritis tended to be later, at 1, 19, and 21 months. The present case is also seronegative, with a late onset of symptoms at 22 months. It is suggested that the seroreactivity may be related to the time of onset. In several ICI‐associated rheumatoid arthritis cases, the response to rheumatic symptoms is good post‐ICI discontinuation or steroid administration. Our patient also showed an immediate response to treatment and remission, suggesting that rheumatoid arthritis is an irAE for which remission is easily achieved. However, bone destruction has been reported within a few months of the onset of symptoms, and irreversible functional impairment may occur.^6^ There have been reports of irAE relapse after ICI resumption or during steroid reduction, and careful follow‐up is essential after resuming ICI.^7^, ^8^ However, only a few reports have discussed arthritis relapse after resuming ICI. More reports of no symptom relapse after the resumption of ICI or steroid reduction, as in this case, would further contribute to future practice. The differences between seronegative and seropositive rheumatoid arthritis, including the differences between clinical course and response to treatment, are unclear and will need further clarification. Some reports indicate that steroids do not impact ICI antitumor effect, while others suggest that steroids may diminish the effect of ICI and lead to cancer progression.^9^, ^10^ In this case, the metastases were a stable disease, but the metastatic lesions were residual. The antitumor effect of steroids may cause the tumor to grow in the future; therefore, we decided that ICI should be restarted. Steroid therapy in patients receiving ICI may be detrimental and should be considered individually.

**Table 1:** Present and previous reports of rheumatoid arthritis including seronegative rheumatoid arthritis that developed during ICI therapy

Author	Patients (no.)	Age (years)	Gender	Disease	ICI	Onset of symptoms after initial ICI administration	Initial treatment for RA	Discontinuation of ICI	Therapeutic course	Readministration of ICI	Symptom relapse after readministration	Tumor response	Autoantibody result	Classification
Belkhir^11^	1	55	Female	Squamous cell carcinoma of the vagina	Nivolumab	1 month	NSAIDs	Yes	Resolution	Yes	NR	Progressive disease	CCP: 671 U/mL RF: 18 IU/mL	Seropositive
Belkhir^11^	2	66	Female	Endometrial adenocarcinoma	Pembrolizumab	1 month	PSL 10 mg/day	Yes	Resolution	Yes	NR	Stable disease	CCP: 233 U/mL RF: 180 IU/mL	Seropositive
Belkhir^11^	3	59	Male	Lung adenocarcinoma	Nivolumab	4 month	PSL 10 mg/day	Yes	Resolution	Yes	NR	Partial response	CCP: 61 U/mL RF: 47 UI/mL	Seropositive
Belkhir^11^	4	56	Female	Metastatic melanoma	Pembrolizumab	1 month	NSAIDs + HCQ 400 mg/day	Yes	Good response	Yes	NR	Stable disease	CCP: 18 U/mL RF: <15 IU/mL	Seropositive
Belkhir^11^	5	80	Male	Metastatic melanoma	Nivolumab	1 month	PSL 15 mg/day + HCQ 200 mg/day	Yes	Good response	Yes	NR	Stable disease	CCP: 42 U/mL RF: <15 UI/mL	Seropositive
Belkhir^11^	6	68	Male	Lung adenocarcinoma	Nivolumab	1 month	NSAID: no effect stopping nivolumab and MTX 10 mg/week	Yes	Good response	No	NR	Stable disease	CCP: >300 U/mL RF: 246 IU/mL	Seropositive
Le Burel^12^	7	66	Female	Endometrial adenocarcinoma	PD1	1 month	Corticosteroid 25 mg	NR	Good response	NR	NR	NR	CCP+ RF+	Seropositive
Le Burel^12^	8	54	Female	Vaginal squamous cell carcinoma	PD1	1 month	Corticosteroid 10 mg	NR	Good response	NR	NR	NR	CCP+ RF+	Seropositive
Le Burel^12^	9	60	Male	Lung adenocarcinoma	PD1	4 month	Corticosteroid 10 mg	NR	Resolution	NR	NR	NR	CCP+ RF+	Seropositive
Hasegawa^13^	10	63	Male	Lung adenocarcinoma	Nivolumab	5 month	Steroid	Yes	Good response	Yes	NR	Progressive disease	NR	Seropositive
Tomizawa^14^	11	67	Male	RCC	Nivolumab(3 mg/kg/2 week)	4 month	PSL 5 mg/day + MTX 6 mg/day SASP 500 mg/day	Yes	Good response	No	NR	Stable disease	CCP: <0.6 U/mL RF: 21 IU/mL	Seropositive
Kwok^15^	12	36	Male	Oropharyngeal squamous cell carcinoma	Nivolumab (3 mg/kg/2 week)	6 month	MTX 10 mg → hydroxychloroquine	NR	Good response	NR	NR	NR	CCP− RF: 16 IU/mL	Seropositive
Verspohl^16^	13	69	Female	Gastrointestinal tumor	Nivolumab	0.5 month	glucocorticosteroid + MTX	NR	Good response	NR	NR	NR	CCP+ RF+	Seropositive
Verspohl^16^	14	72	Male	Metastatic melanoma	Pembrolizumab	21 month	Glucocorticosteroid + MTX	NR	Good response	NR	NR	NR	CCP− RF−	Seronegative
Verspohl^16^	15	71	Female	Metastatic melanoma	Pembrolizumab	1 month	Glucocorticosteroid + tocilizumb	NR	Good response	NR	NR	NR	CCP− RF−	Seronegative
Haikal^17^	16	65	Female	Melanoma	Nivolumab	19 month	Low‐dose prednisone + hydroxychloroquine → MTX + leflunomide	NR	Resolution	Yes	No	NR	CCP− (<17 U/mL) RF− (<14 IU/mL)	Seronegative
Present case	17	80	Male	Reanal cell carcinoma	Ipilimumab (1 mg/kg) + nivolumab (240 mg) (4 cycles) then nivolumab only (240 mg/2 weeks)	22 month	PSL 20 mg	Yes	Resolution	Yes	No	Stable disease	CCP: 0.6 U/mL RF: <10 IU/mL	Seronegative

## Conclusion

We herein report a case of seronegative rheumatoid arthritis that developed during treatment with ipilimumab‐nivolumab for metastatic RCC. Seronegative rheumatoid arthritis should be included in the differential diagnosis when arthritis develops during ICI treatment. It is suggested that seronegative rheumatoid arthritis due to ICI can be easily remitted by ICI interruption and steroid administration, as in this case.

## Author contributions

**Yuki Nishimura:** Data curation; investigation; visualization; writing – original draft. **Kazuaki Yamanaka:** Conceptualization; project administration; writing – review and editing. **Taigo Kato:** Writing – review and editing. **Koji Hatano:** Writing – review and editing. **Atsunari Kawashima:** Writing – review and editing. **Shinichiro Fukuhara:** Writing – review and editing. **Motohide Uemura:** Writing – review and editing. **Ryoichi Imamura:** Writing – review and editing. **Norio Nonomura:** Writing – review and editing.

## Conflict of interest

The authors declare no conflict of interest.

## Approval of the research protocol by an Institutional Reviewer Board

Not applicable.

## Informed consent

Informed consent was obtained from the patient for publication of the case particulars.

## Registry and the Registration No. of the study/trial

Not applicable.
